# A Survey on Online Political Participation, Social Capital, and Well-Being in Social Media Users—Based on the Second Phase of the Third (2019) TCS Taiwan Communication Survey Database

**DOI:** 10.3389/fpsyg.2021.730351

**Published:** 2022-01-03

**Authors:** Fangqi Zhong, Pengpeng Li, Jinchao Xi

**Affiliations:** ^1^College of Communication, National Chengchi University, Taipei, Taiwan; ^2^Graduate Institute of Development Studies, National Chengchi University, Taipei, Taiwan

**Keywords:** social media, internet political participants, happiness, social capital, SEM

## Abstract

This study focused on the frequency of social media use. Through investigating and verifying the correlations between social media use frequency, online political participation, and social capital, we derived two models of socialization that affect citizen well-being and, accordingly, proposed strategic suggestions for democratic society construction and network management. This study drew upon the 2019 Taiwan Communication Survey database and used structural equation modeling (SEM) as a statistical method to explore the causal relationship between these four variables (social media use, online political participation, social capital and well-being). The data analysis yielded an overall good fit with the overall fit indicators: χ^2^ = 214.417, *df* = 84, *p* = 2.293, RMSEA = 0.028, GFI = 0.998, CFI = 0.986, SRMR = 0.066, and *CN* = 993.411. Future communication scholars who wish to explore issues related to new media users can draw on this model for subsequent research.

## Introduction

Social media is a popular online platform for public sharing, creation, and communication. Its instantaneous, interactive, and borderless nature sets it apart from traditional media. Social media has not only changed the way the people communicate, but has also become an increasingly important channel for the dissemination and discussion of socio-political issues, which, in turn, has had a profound impact on the course of political mediatization in democratic countries. Through virtual networks, social media platforms can provide the communication and information facilities to allow citizens to participate in public discourse and criticism of laws or policies. This potential may help to build substantive democratic institutions, thereby strengthening democracy in general, and help governments and political parties to access and develop public trust (Saud and Margono, 2021). Pew Research Center (2012, 2014) reports that more than 30% of social media users have expressed their views or opinions on political and social issues on social media platforms. In addition, people who frequently use social media and follow related political figures or topics show more willingness to participate in socio-political public affairs. In terms of motivation for civic participation, more and more young people are involved in democratic, social, and political activities, and consider this involvement a basic part of a responsibility to contribute to their country (Saud et al., 2020a). Citizen participation in political public affairs, as well as the process of communicating and gaining acceptance from others, can produce psychological well-being (PWB) and satisfaction (Parry et al., 1992). Thus, finding ways to enhance positive political participation and life well-being index is important to consider in improving the online communication mode for socio-political issues and, by extension, building a democratized nation.

In *Social Network Theories*, Mark Granovetter (1973) proposed “The Strength of Weak Ties”: his research found that people’s “weak ties” in society are not as strong as their “strong ties,” but have the advantage of allowing instant, low-cost, and efficient communication. In modern society, social networks such as Facebook, Twitter, and microblogs are used as intermediaries to form a kind of “weak connection” between people, which accelerates and broadens information spread and, at the same time, allows geographically and socially distant strangers to meet, aiding the establishment and growth of new weak connections. It is beneficial to strengthen the spirit of information sharing in society; people can develop the positive emotions of trust and mutual support through communication and interaction in the network, and implicitly accumulate personal social capital (Coleman, 1988; Kobayashi, 2010). Moreover, human beings have a need to live in groups and interact with each other; psychological and spiritual enrichment and satisfaction often need to be achieved through mutual interaction, and personal values are usually realized and enhanced in well-functioning social networks (Ye, 2006; Valenzuela et al., 2009). In addition, Lin’s (2017) study pointed out that social media use extends interpersonal connections, strengthens social relationships/capital, and even promotes positive interactions among users, enhancing positive feelings and improving quality of life, self-satisfaction and well-being. Therefore, having the social capital to make good use of is an indispensable element for a person to have a happy and beautiful life.

In summary, this study focuses on the frequency of social media use, and through investigating and verifying the correlation between the frequency of social media use and citizens’ political participation and social capital accumulation, two models of socialization that affect citizens’ well-being are derived, and strategic suggestions for democratic society construction and network management are proposed accordingly.

## Review of the Literature

### Frequency of Social Media Use and Online Political Participation

Social media is a virtual internet community platform upon which people create, share, and exchange opinions, views, and experiences (Saud et al., 2020b). Because of the characteristics of social media (highly interactive, easy to operate, open resources, can be shared anytime and anywhere), participation in political or public affairs discussion has become significantly easier: people are able to express their personal views more directly, freely, and openly, making the communication between administration, organizations, and people more convenient and smooth (Kim and Hoewe, 2020).

Political participation can be expressed as “the act of citizens participating in or influencing the political and public affairs agenda” (Van Deth, 1986; Kim and Hoewe, 2020); however, there is no consistent definition in academic circles. Previous studies on political participation have focused on the forms of participation and the impact on political movements or public affairs, as well as on traditional modes of political participation such as voting, demonstration, participation in political rallies, and donations (Van Deth, 2016). However, with the coverage and popularity of the internet, the modes of civic political participation have diversified, and can now take the form of emails to politicians, visiting campaign websites, and making donations online (Gibson et al., 2005). In their study, Kim and Hoewe (2020) discuss and summarize the five main types of civic political participation: (1) traditional political participation; (2) interpersonal political discourse; (3) voting; (4) social media participation; and (5) online information search. Clearly, internet-based political participation is gradually becoming a primary arena for the fight for rights and social development.

Putnam (2000) suggests that social media use may promote the most important civic consciousness in a democratic society and equip government institutions and leadership with greater transformative democratic sensitivities. Regular users of social media receive more information about political and public news (Ida et al., 2020), and people who share views, through discussion, are able to increase their engagement and understanding of issues and, consequently, their motivation to participate in political and public affairs (Eveland, 2004; Kim and Chen, 2016). Elin (2013) also points out that social media provides a space where individuals can engage in politics and generate political action: in short, when individuals use social media and access information, they are more inclined to participate in politics (Chen, 2016). Most of the aforementioned literature has validated the ability of social networks to provide citizens access to political information. This access (specifically through social networks) is positively correlated with online political participation. However, these papers do not address the effect of social media usage frequency (e.g., self-exposure, post sharing, and browsing frequency) on citizens’ online political participation: there is still a significant need for further discussion and testing of this concept. Accordingly, this study proposes the following hypothesis:

H1: Frequency of social media use and online political participation are positively correlated.

### Frequency of Social Media Use and Social Capital

The concept of social capital originated in community-centered research in rural schools, with particular emphasis on concepts such as morality, friendship, empathy, and social interaction. Bourdieu (1986) was the first to introduce the concept of social capital into sociology, defining it as “the sum of actual or potential resources linked to group membership,” which are associated with the possession of an enduring network of mutual acquaintanceship and institutionalized relationships, that is, relationships with the membership of the group, which provides the capital backing to its members.

However, as a concept, social capital is still difficult to define and measure operationally due to its controversial status in the academic community (Pooley et al., 2005). In general, social capital is divided into three levels: micro, meso, and macro (Brown, 1997). Micro social capital consists of views embedded in the self, such as personal ties to family heritage and geography, which exist in the form of relationships. Individuals can obtain the required resources such as information, job opportunities, knowledge, influence, social support, and long-term social cooperation through these social relationships. The meso level focuses on the impact of organizations’ network structures on social capital from the perspective of social network structure, which exists in the form of informal institutions, organizational practices, and customary rules, and emphasizes the resource availabilities for individuals, firms, communities, groups, etc., allowed by their specific position in the social structure. The macro-level view of social capital, also known as embedded structure, is concerned with the appropriation of social capital by a particular group of actors in an organization, society, or state, and includes harmonious networks of social relations, effective institutional norms, and general trust. Social capital, at the micro and meso levels, is collectively referred to as “bridge social capital” because it arises from the external social relations of actors and functions to help these actors access external resources. Macro social capital, on the other hand, is called “bonding social capital” because it arises from relationships within the group and functions to enhance the collective action of the group or organization (Adler and Kwon, 2002).

While social media use’s impact on social capital receives increasing academic attention, the general influence of social media on social capital is still debated. In the past, face-to-face human interactions were central to sustaining social capital. However, recent research has highlighted a positive relationship between social media use and social capital (Gil de Zúñiga et al., 2012; Brown and Michinov, 2019; Tefertiller et al., 2020). Current scholarship suggests that the internet is associated with either an increase or decrease in social capital (Ellison et al., 2007); optimists argue that many internet users generate face-to-face interpersonal interactions *via* video (Kraut et al., 1998). Therefore, internet use may, in fact, increase interpersonal interactions (Nie, 2001) and thus have a positive effect on social capital (Ostic et al., 2021). Conversely, pessimists argue that internet use takes up people’s time and therefore reduces time available for face-to-face interactions (Putnam, 2000). Accordingly, this study posed the following research question:

RQ1: What is the relationship between frequency of social media use and social capital?

### Networked Social Sources of Well-Being

Currently, there are two major schools of thought surrounding definitions of well-being, namely subjective well-being (SWB), which is based on the philosophical view of well-being, and PWB, which is based on the philosophical view of realism and studies human potential. PWB places emphasis on theoretical constructs—exploring well-being from a theoretical perspective—and its structure is proposed and validated based on theoretical foundations (Zhang and Zuo, 2007). The study of PWB can provide a more comprehensive understanding of the meaning of well-being; therefore, this study employed the PWB definition.

First, past research has demonstrated a positive relationship between citizens’ political participation and levels of well-being. Regarding participatory behavior, studies on voting in Switzerland have demonstrated that people with more opportunities to participate in the democratic process are more satisfied with their lives than those with less, and that Swiss citizens in cantons with higher opportunities to participate have significantly higher levels of well-being (Cacioppo and Patrick, 2008). Canadian electoral studies have also demonstrated that the primary factor increasing personal satisfaction is electoral voting behavior rather than actual election outcome. Simultaneously, people with higher levels of well-being are more likely to participate in offline electoral voting activities (Goodman, 2014). Wang (2014) argues that citizens have a latent desire to participate in political activities in a democratic political system, and that in the process of asserting civil rights and being recognized by society and the state, their value as participants in public affairs is recognized and respected. In other words, the opportunity to participate in political or social public affairs offers citizens’ the opportunity to fully express their political desires and demands for power. However, all the above-mentioned studies focus on the correlation between civic participation and well-being; few studies have discussed whether social media use can enhance personal well-being through active social media–based political participation. It is also important to note that early studies on social media use have tended to examine its positive psychological effects (Ellison et al., 2007; Steinfield et al., 2008); e.g., higher social media use frequency is associated with higher perceived social capital, perceived social support, and PWB (well-being). However, the internet’s increasing popularity has made the negative effects of social media usage (such as cyber-addiction, cyber-bullying, and privacy breaches) more apparent. Therefore, this study does not intend to explore the direct relationship between social media frequency and well-being, but rather focuses on whether political participation can enhance citizens’ well-being in the social media context and, accordingly, proposes the following hypothesis and research question:

H2: Degree of online political participation in the community is positively associated with well-being.

RQ2: Is online political participation a mediating variable between frequency of social media use and well-being?

Second, in virtual social networks, people can generate the positive feelings of trust and mutual support through free communication and interaction, both of which are important for well-being (Chen M. H., 2014). At the micro level, social capital is a resource embedded in a social structure; an individual’s ability to access social resources; and the ability to use or mobilize these social resources through purposeful actions that enable individuals to receive financial, political, and social rewards, and other benefits such as physical health, mental health, and life satisfaction (Lin, 2002). At the macro level, social capital promotes economic growth, a positive outlook for the future, and increased life satisfaction (Zak and Knack, 2001). Social media use has been generally shown to help extend one’s social network and strengthen social relationships, which in turn promotes positive interactions between users in general and enhances positive psychological perceptions, quality of life, and self-satisfaction (Ellison et al., 2006, 2007; Amichai-Hamburger and Furnham, 2007; Furnham and Christoforou, 2007; Neale and Russell-Bennett, 2009; Valenzuela et al., 2009). Overall, social capital has a direct positive impact on well-being from an individual, social, and national perspective (Chen M. H., 2014), and social media use can thus increase individual well-being through personal social capital accumulation (Docherty, 2020). Accordingly, this study proposes the following research hypotheses:

H3: There is a positive association between social capital and well-being.

H4: Social capital is a mediating variable between frequency of social media use and well-being.

In summary, past studies have confirmed that the frequency of social media use affects online political participation, and that political participation and civic well-being are related. It can also be concluded from our review that social capital in the network is also related to civic well-being; however, there is a lack of knowledge about causality, mediating effects, and overall model fit regarding the two social channels that influence civic well-being in social media contexts, i.e., political participation and social capital. Therefore, this study intends to investigate the structural relationship between the four factors, using the above empirical study and theory as a basis for constructing and validating the structural model of social media usage frequency, online political participation, social capital, and well-being.

## Research Methodology

### Sources of Information

This study used data collected from the 2nd phase of the Taiwan Ministry of Science and Technology Communication Survey Database (2019) for secondary data analysis. For this study, the main advantages of this method are: (1) convenience, i.e., the researcher does not need to spend a lot of money and time to analyze data already provided by a large sample; and (2) the database’s data collection is usually based on the principle of random sampling, allowing this study to extrapolate its results to wider society.

In this survey, multi-stage stratified random sampling was used in the following stages: (1) sampling of villages, towns, and urban areas; (2) sampling of minimum enumeration areas; (3) sampling of door numbers; and (4) sampling of households according to age (see Chang, 2020).

### Sample Profile

The survey was conducted on the theme of “media use and social interaction.” Face-to-face interviews were conducted with people who had household registration in Taiwan, were over 18 years old, and lived at the sample address at least 4 days a week.

The interviews were conducted from 4 July 2018 to 11 October 2018, and a total of 2,028 valid samples were collected. Regarding sample structure, the distribution of gender, age, marital status, and educational attainment was similar to that of past demographic and communication survey databases, achieving representativeness and inferential generality. Specifically, there were 998 (49.2%) male respondents and 1,030 (50.8%) female respondents in the sample. The age distribution was mainly 20–69 years old and relatively evenly distributed: 30–39 (18.8%), 40–49 (18.7%), 50–59 (18.6%), 20–29 (16.2%), 60–69 (14.4%), 70+ (10.5%), and 18–19 (2.8%). Most respondents were married (1,227; 60.5%), followed by 592 (29.2%) who were unmarried, 117 (5.8%) whose spouses had died, 79 (3.9%) who were divorced or separated, 11 (0.5%) who were cohabiting, and 1 (0.1%) who was otherwise. Education level was dominated by university (505; 24.9%), high school (high business, high engineering) (332; 16.4%), national (junior) high school (239; 11.8%), and primary school (224; 11.1%).

### Definition and Measurement of Variables

A potential preliminary structure connecting social capital, online use, and political participation to well-being is evident in the literature, but there exists a lack of quantitative research on it. To begin to explore this relationship, this study, based on research motivation, research questions, and the results of related literature analysis, defines its main variables: Facebook usage, social capital, online political participation, and well-being.

#### Variables—Frequency of Social Media Use

In this study, the four items in the Communication Survey Database questionnaire were used to construct the variable scale (*SD* = 0.87); (1) How often do you post messages on social media? (*M* = 2.45, *SD* = 0.86); (2) How often do you share or repost messages on social media? (*M* = 3.28, *SD* = 0.76); (3) How often do you look at messages posted by your friends on social media? (*M* = 3.37, *SD* = 0.76); (4) How often do you look at messages shared or reposted by your friends on social media?( *M* = 3.37, *SD* = 0.71).

For the four questions of the scale, Bartlett’s test of sphericity was significant (*X*^2^ = 2279.43, *df* = 6, *p* < 0.001), and the KMO sampling adequacy value was 0.57, which was not suitable for principal component factor analysis; however, the overall reliability of the scale was quite good (Cronbach’s α = 0.75).

#### Mediator Variables

##### Online Political Participation

Online political participation is a part of civic engagement, and its main behaviors, such as forwarding political information, are aimed at influencing government actions and policy making (Verba et al., 1995). This variable was constructed from a database of three questions, using a 4-point scale that allowed participants to circle the response according to their own situation (1 = never, 2 = rarely, 3 = sometimes, 4 = often): (1) How often do you search, browse, click, or watch news, messages, or videos related to politics and public affairs on online platforms? (*M* = 2.61, *SD* = 0.96); (2) How often do you share and repost news, messages, or videos related to politics and public affairs on online platforms? (*M* = 2.57, *SD* = 0.93); (3) How often do you post, produce, leave comments, and comment on news, messages, or videos related to politics and public affairs on online platforms? (*M* = 2.70, *SD* = 0.80).

The Bartlett’s sphericity test for the three questions reached significant levels (*X*^2^ = 94.58, *df* = 3, *p* < 0.001), with a KMO sampling fitness value of 0.67, allowing for principal component analysis, where only one factor was extracted between questions using the rule of eigenvalue greater than one, cumulatively explaining 72.90% of the total variance. At the same time, the negative factor loadings of all three questions of the scale were greater than 0.7, which had very high convergent validity; the overall scale had very good reliability (Cronbach’s α = 0.81).

##### Social Capital

Different types of social capital have been classified according to different social network conditions. Based on the above-mentioned literature, this study adopts “bridging social capital” and “bonding social capital” as the two components of the concept of “social capital,” and employs the social capital scale developed by Williams (2003). “Bridging Social Capital” selected three items and extracted data from a 5-point agreement scale based on these items: (1) Contacting with other people makes you want to try new things? (*M* = 3.46, *SD* = 0.95); (2) Contacting with other people makes you want to try new friends? (*M* = 3.29, *SD* = 0.99); (3) Small talking with others makes you curious about what is happening in the world (*M* = 3.45, *SD* = 0.96).

Three items were selected for “Fitting Social Capital,” and subjects were asked to indicate level of agreement with each according to a 5-point scale: (1) When you are in trouble, you can find someone you trust to help you solve your problems (*M* = 3.90, *SD* = 0.77); (2) When you have a private problem, there is someone you can talk to safely (*M* = 3.90, *SD* = 0.78); and (3) When you feel lonely, you can find someone else to talk to (*M* = 3.93, *SD* = 0.76).

The Bartlett’s sphericity test for the six items on this scale was significant (*X*^2^ = 6036.67, *df* = 15, *p* < 0.001), with a KMO sampling suitability value of 0.74. For principal component analysis, using the eigenvalue greater than one rule, two factors were extracted between the questions, cumulatively explaining 78.90% of the total variance. By using orthogonal rotations (varimax), the factor loadings of all six items were greater than 0.7, with high convergent and discriminant validity, and the scale showed “bridging social capital” and “bonding social capital,” these two dimensions obviously. At the same time, the item analysis revealed, the three questions of “bridging social capital” (Cronbach’s α = 0.84) and the three questions of “binding social capital” (Cronbach’s α = 0.88) were found to have good reliability.

#### Dependencies—Well-Being

The six common components of PWB were summarized by Ryff (1989). and confirmed through empirical research which defined PWB in both theoretical and operational terms. Among these six dimensions, according to Ryff (1989), the pursuit of life goals and having harmonious interpersonal relationships are the two most important factors for a person’s well-being, followed by respect for and mastery of self.

Five questions were selected from the database to construct the variable scale, and subjects were asked to rate their agreement on a 5-point scale: (1) Overall, are you satisfied with your life? (*M* = 3.73, *SD* = 0.74); (2) Overall, are you satisfied with your work? (*M* = 4.24, *SD* = 1.23); (3) Overall, are you satisfied with your social life? (*M* = 3.78, *SD* = 0.69); (4) Overall, are you satisfied with your health? (*M* = 3.56, *SD* = 0.84); and (5) Overall, are you satisfied with yourself? (*M* = 3.71, *SD* = 0.72).

The Bartlett’s sphericity test for the five questions of the scale was significant (*X*^2^ = 2721.87, *df* = 10, *p* < 0.001), with a KMO sampling suitability value of 0.77. For principal component analysis, using the rule of eigenvalue greater than one, only one factor was extracted between questions, explaining 52.86% of the total variance cumulatively. The factor loadings of all five questions were greater than the minimum standard 0.5, with convergent validity, and the overall scale had good reliability (Cronbach’s α = 0.72).

#### Control Variables—Gender

Statistical analysis was used to control for variables that may affect the dependent variable in order to exclude them as much as possible. Typically, Pearson’s correlation coefficient is between −1 and 1, meaning there is a covariate between two variables and, according to Cohen’s (2013) definition of the effect size of the correlation coefficient (r), this effect is small when *r* ≥ 0.1, medium when *r* ≥ 0.3, and large when *r* ≥ 0.5. If the correlation coefficient d between covariates and dependent variables is greater than 0.2, the use of covariate analysis can improve the explanatory power of the results; however, if the correlation coefficient is less than 0.2, covariate analysis is not as effective as the results of simple variance analysis (Keppel and Zedeck, 1989). According to the existing theoretical literature, gender, age, education level, interest in political issues, ideology, frequency of political discussion, social network size, and network diversity all have an impact on the dependent variable “well-being” in this study. However, since this study is a secondary data analysis, the observable variables are limited by the original questionnaire designs, and the variables that can be selected to influence the dependent variable are gender, age, education level, interest in political issues, and ideology. The correlation test found that gender had the most significant effect on the dependent variable (*r* = 0.44^***^, *p* = 0.00). In addition, the two variables with high correlation coefficients could not be statistically analyzed as covariates at the same time. The age of the subjects (*r* = 0.31^***^, *p* = 0.00), education level (*r* = 0.22^***^, *p* = 0.00), interest in political issues (*r* = 0.18^***^, *p* = 0.00), and ideology (*r* = 0.27^***^, *p* = 0.00) were also correlated with the dependent variable, but their effects on the dependent variable were not as strong as that of subject gender; accordingly, “gender” was used as the only covariate in this study.

### Statistical Methods and Data Processing

Since the frequent assumption of statistical methods (such as traditional path analysis) that observed variables are completely reliable and free of errors is rarely true in real-world situations, it is essential to fully consider and examine the observed variables (questions) within the potential variables. At the same time, the regression analysis of traditional statistical methods can reduce the significance level of each variable due to multiple independent determinations when testing the correlation of multiple variables. Structural equation modeling (SEM) is an integrated research technique that “measures” the error of observed variables and “analyzes” the causal relationship of potential variables (Qiu, 2005; Gefen et al., 2011). Its main function is to explain the relationship between the structure of potential factors and the observed variables, as well as the interaction effects between the observed variables (Chen X. F., 2014). This method of statistical analysis is gradually gaining recognition among researchers for its convenience and explanatory validity; it can be used for multiple independent and dependent variables simultaneously and can produce results for all regression coefficients at α = 0.05. Therefore, this study aims to perform factor analysis, path analysis, and causality analysis on the experimental questionnaires, using the structural equation model to scrutinize and understand the relationship between each potential independent variable and potential dependent variable, as well as the specific explanatory power of the observed variables within each potential variable.

We first determined the structure and direction of this study based on the results of the literature, then administered the test, and then conducted a statistical analysis of the data using JASP. In the analysis of the data, firstly, a confirmatory factor analysis (CFA) method was used in the SEM to explore the measurement model fitness of the four variables under the premise of controlling the “gender” of the independent variables, until the model was adjusted to the best fit. Next, a path analysis between the potential variables was conducted for the structural model, and a further path analysis was then conducted for the structural model to test the fitness of the path relationships among the potential variables. The above analysis was estimated using a pre-determined maximum likelihood estimation (MLE), and the overall fitness metric, the basic fitness metric, and the intrinsic fitness metric were used as the basis for checking the fitness of the proposed model in this study (Yu, 2006; Kline, 2015).

## Analysis of Information

**FIGURE 1:**
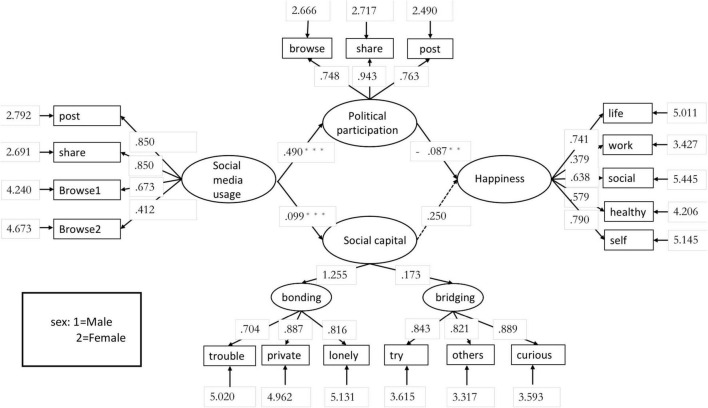
A measurement model of the relationship between frequency of social media use, online political participation in the community, social capital, and well-being. *** means *p* < 0.001, **means *p* < 0.01.

The results of the measurement model showed that χ^2^ = 1632.903, *df* = 129, *p* < 0.001, although GFI = 0.993, which has a good degree of fit with the observed data (Howell, 1996). However, RMSEA = 0.076 and CFI = 0.872, indicating a poor fit (Hu and Bentler, 1999), means there were factors that affect the model fit. Specifically, there were items with the factor loading coefficient less than 0.7 in scales, as shown in Figure 1.

**FIGURE 2:**
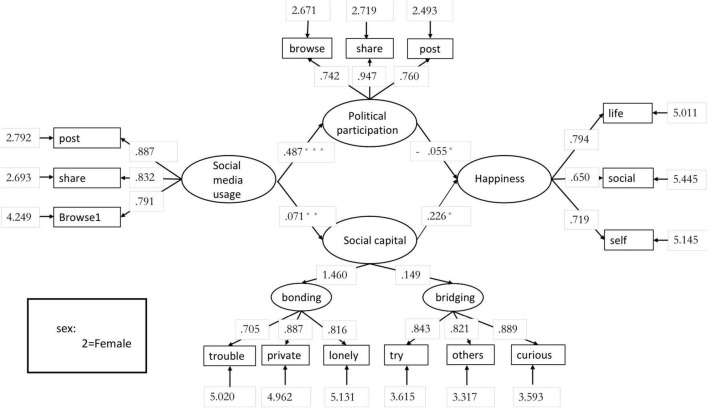
Structural equation model of the relationship between frequency of social media use, online political participation, social capital, and well-being. *** means *p* < 0.001, **means *p* < 0.01, *means *p* < 0.05.

As can be seen from the above figure, after controlling for the “Gender” variable, “Browse2” in the “Social media usage” variable, and “work” and “healthy” in the “Well-being” variable, did not meet the minimum factor loading criterion (>0.6). “Work” and “healthy” in “Well-being” were thus removed. The deleted structural equation model showed that χ^2^ = 214.417, *df* = 84, and *p* = 2.293, which is greater than 0.05 and does not reach statistically significant difference, indicating that this model has good explanatory power. RMSEA = 0.028, which meets the rubric of fitness, i.e., this model is a reasonable fit; GFI = 0.998, which shows that this model has a good degree of fit with the observed data (Howell, 1996); CFI = 0.986, indicating a good improvement compared to the independent model without covariance; and SRMR = 0.066, indicating a good fit (Hu and Bentler, 1999). For this model, the streamlined normative fitness index *CN* = 993.411, which is greater than 200, shows that this model is good in parsimony. See Figure 2 for details.

Next, the research hypotheses were tested separately, based on the results of the SEM.

(1) There is a significant positive correlation between frequency of social media use and online political participation (H1 holds); its effect value reached 0.47 (*t* = 2.37, *p* < 0.001), i.e., the more frequently people post and share content posted by their friends in social networking platforms, and read messages shared or reposted by their friends, the more they are involved in political and public affairs in the internet world. (2) The degree of political involvement in social networking is negatively related to PWB (H2 does not hold); its effect value was 0.21 (*t* = 1.91, *p* < 0.05), i.e., the more often people participate in political discussions and share relevant information in social networking platforms, the less positive feeling about their life, socialization, and satisfaction or well-being; however, this effect was weakly significant (*p* < 0.05). (3) There is a positive relationship between social capital and well-being (H3 holds); the effect value was 0.23 (*t* = 1.99, *p* < 0.05), i.e., when people have more intimate interpersonal resources and form a tighter social network, they will have more positive and optimistic attitudes toward life in general and be more satisfied with their own evaluations (this finding is reflected in past studies on social capital). (4) H4 mainly relied on verifying the mediating effect. In recent years, bootstrapping has often been used to validate mediation effect; it can be used to obtain more detailed statistical data to predict the parent expectation, distribution, and interval by re-sampling the original sample (in this study, 5000 times) with the statistical assumption of near infinite sampling. This study used JASP bootstrapping to test the mediating effect proposed by H4, showing that, after controlling for gender variables, the frequency of social media use significantly affects citizens’ well-being positively through social capital accumulation in social networks. In other words, social capital mediates the positive effect of frequency of social media use on well-being (*B* = 0.03, *SE* = 0.02, 95% confidence interval: 0.13–0.19, *p* < 0.01).

For RQ1 (“What is the relationship between frequency of social media use and social capital?”), the results of the analysis showed a strong positive correlation between the two potential variables; the effect value was 0.51 (*t* = 3.17, *p* < 0.01), indicating that with development of internet and user-side technology, people’s social interactions are gradually moving online. In the process of using online social platforms, people interact with their friends by browsing, retweeting, and posting, which in turn leads to better trust, intimate interpersonal relationships, and curiosity about different things, and leads to the accumulation of social capital. For RQ2 (“Is online political participation a mediating variable between frequency of social media use and personal well-being?”), it was found that, after mediation effect analysis and controlling for the gender variables, the frequency of social media use had a relatively weak negative effect on civic well-being through citizens’ political participation in social networks. That is, online political engagement mediated the negative effect of social media usage frequency on well-being (*B* = 0.14, *SE* = 0.04, 95% confidence interval: 0.27 –0.33, *p* < 0.05).

## Conclusion and Discussion

This paper tests a structural model of frequency of social media use, political participation, social capital, and well-being. The results of the data analysis can be summarized on two aspects.

First aspect, the factors influencing civic well-being from the perspective of social media usage frequency and online political participation can be summarized into three claims: (1) The more often users use social media to post, share, and view the content of others’ messages, the more willing they are to view, retweet, and post news and videos on social platforms in order to participate in political and public affairs (H1). This view is consistent with the past finding that frequency of social media use is an important variable in influencing citizens’ political participation online and offline (Kim and Khang, 2014). In other words, the more time spent on social media, the higher the level of civic political participation, and participation in political activities helps to enhance citizens’ knowledge and provides them with opportunities to participate and build political capacity (Ida and Saud, 2020).

It can be inferred, then, that in today’s democratic politics and public life, social media occupies an important place in political communication, through which governments can rule and formulate relevant strategies and the public is empowered to understand government administration and social issues. This thesis coincides with the view of mediated politics. In today’s world, mediated politics plays a kind of “intermediary” role in modern society: it centers on political power and government on the one hand, and the will of the people on the other. Politics acts as an agent, relying on its political power to control and regulate public services, health, and other social structures. By using the media to manage public opinion and build bridges with the public, politics brings the public into public political life, providing citizen access to democracy.

(2) Degree of online political participation is negatively correlated with well-being (H2). A possible explanation is that citizens’ online political participation mainly aims at monitoring, expressing demands, and communicating; however, in terms of practical effect, although online platforms give citizens the right to freely express their views, they may not allow citizens’ demands to be satisfied, and citizen–government communication may not substantially alter government decisions. Thus, citizens’ well-being with their life, self, and social life is weakened. What is more concerning is that the internet’s rapid and flattened dissemination of information has turned social media platforms into a complex and volatile sphere of public opinion, where everyone can be a news spokesperson and everyone can be influenced by a mix of truths and falsehoods. Moreover, news contrary to one’s own expectations can be spread rapidly, thus affecting satisfaction and well-being. Accordingly, there exists urgent needs to improve media literacy in the context of information transparency and to establish fact-checking institutions to counteract fake news, to provide a platform for online political participation and promotion of the democratic process.

(3) This study found that frequency of social media use does not have a direct and significant impact on citizen well-being, but rather increases citizens’ well-being with their life in general, social life, and self through the *reduction* of their online political participation (RQ2), or through the accumulation of social capital (H4). On the one hand, increased use of social media has given citizens more opportunities to get involved and participate in political issues and public affairs discussions. However, because of the ambiguous (or even contradictory) relationship between the virtual and the real, users who communicate on this platform are often disturbed by their own emotional communication, and the suspension of technical “empowerment” and actual “power” may lead to negative effects such as secondary public-opinion generation, political-value dislocation, proliferation of moral rhetoric, restricted participation in cognition, and the overstepping of public supervision, which, in turn, weakens citizens’ well-being in real life (Tefertiller et al., 2020). On the other hand, the refinement of social media algorithms and the increase in social media use frequency increase the chances that users come into contact with groups related to their own personality traits, interests, and needs, contributing to the accumulation of personal social capital, which can provide rich social support for the expression and development of personal values, thus enhancing citizen well-being. This, in turn, enhances citizens’ sense of well-being in a group society.

Second aspect, the factors influencing citizens’ well-being from the perspective of social media usage and social capital accumulation can be summarized into two points. (1) The more often citizens post, share and view others’ messages on social media, the more their “bonding social capital” and “bridging social capital” is enhanced. As users connect with friends on social platforms, they tend to become more trusting and build intimate relationships as well as develop curiosity, which embodies the process of strengthening social capital. This benefit may be grounded in social media users’ ability to construct public or semi-public profiles, articulate their social relationships, and navigate the particular nature of these connections in virtual spaces. In other words, because social media changes the nature of media and the structure of social connections, it may change the very distribution of social relations and the capital embedded in them. It has been suggested that social media possesses the novel characteristics of expressing weak connections, providing self-centered semi-public dialogue, and transforming potential social values into new ways of behaving to change the structure of social communication (Appel et al., 2014). Therefore, the social capital created on social media is not necessarily different from that in offline interpersonal relationships. However, due to database limitations, this study was not able to explore possible differences in the correlations between social media use frequency and online and offline social capital. First-hand statistical data on this topic could be collected and analyzed in future.

(2) This study found that the social capital (“bonding social capital” and “bridging social capital”) accumulated by citizens through social platforms can enhance their life, social life, and self-satisfaction (H3). This finding is different from that of Trepte et al. (2015), who suggested that only the social support or social capital received from offline social interaction can influence citizens’ satisfaction with their lives. It is possible that, in virtual social communication platforms, people deliberately hide their flaws and shortcomings and are thus more able to focus on purposeful needs such as building connections and maintaining social relationships. Moreover, influenced by social media algorithms, users are more likely to see the personal posts and other information content that align with their interests, hobbies, and personality traits, which helps them obtain effective social capital quickly and easily, satisfying their immediate needs and improving their well-being index. In addition, the literature suggests that economic factors such as personal status, occupation, and income are important for well-being (Stevenson and Wolfers, 2008). However, as the standard of living increases, economic factors are no longer the only factors that influence well-being. At this point, non-economic factors, especially concepts related to social capital, tend to be strongly associated with life satisfaction. This can be explained from the perspective of Maslow’s hierarchy of needs, which states that, after satisfying survival needs such as food, water, air, sleep, and security, individuals will turn to social needs, i.e., the need for friendship, love, and affiliation. From a social capital perspective, when an individual has strong social relationships and social networks, they will have more social support in the form of, for example, marriage partners, family, friends, neighbors, workplace social relationships, etc., which, in turn, will generate sufficient capacity to resist negative emotions (Kobayashi, 2010).

In summary, the results of this study demonstrate that the frequency of social media use affects online political participation and social capital generation. Moreover, social media use affects citizens’ well-being with their lives, socialization, and selves through two socialization paths: online political participation and social capital generation. Previous studies have failed to elucidate structural relationships among these four variables. Therefore, this paper designed a model of social media usage frequency, online political participation, social capital, and well-being, and verified the model’s suitability through the model fitness index, which is an innovative contribution in terms of research orientation and democratic-society construction and management.

Knowledge about the political and social pathways affecting citizens’ well-being could be further supplemented in the future by qualitative interviews, which might explain the results of this quantitative study from a hermeneutic perspective. These interviews could also help discuss how citizens’ online political participation reduces well-being in given social contexts. In addition, there is room for continued improvement of the econometric model of the test scale in this study, due to the inherent limitations of secondary data analysis. In future research, it is suggested that first-hand data be added to modify the measurement model and improve the measurement instrument and model construction. Then, the improve model could be compared with the findings of this study.

## Data Availability Statement

Publicly available datasets were analyzed in this study. This data can be found here: https://www.crctaiwan.nctu.edu.tw/.

## Author Conbutions

FZ: evolution of overarching research goals and aims, development or design of methodology, application of statistical, preparation, and creation of the published work, specifically writing the initial draft. PL: development or design of methodology, improved of models, application of statistical, and revising the manuscript critically for important intellectual content. JX: ideas, data filtering and cleansing, revising the manuscript critically for important intellectual content. All authors contributed to the article and approved the submitted version.

## Conflict of Interest

The authors declare that the research was conducted in the absence of any commercial or financial relationships that could be construed as a potential conflict of interest.

## Publisher’s Note

All claims expressed in this article are solely those of the authors and do not necessarily represent those of their affiliated organizations, or those of the publisher, the editors and the reviewers. Any product that may be evaluated in this article, or claim that may be made by its manufacturer, is not guaranteed or endorsed by the publisher.

## References

[B1] Adler P. S. Kwon S.-W. (2002). Social capital: prospects for a new concept. *Acad. Manag. Rev.* 27 17–40. 10.2307/4134367

[B2] Amichai-Hamburger Y. Furnham A. (2007). The positive net. *Comput. Hum. Behav.* 23 1033–1045. 10.1016/j.chb.2005.08.008

[B3] Appel L. Dadlani P. Dwyer M. Hampton K. Kitzie V. Matni Z. A. (2014). Testing the validity of social capital measures in the study of information and communication technologies. *Inform. Commun. Soc.* 17 398–416. 10.1080/1369118X.2014.884612

[B4] Bourdieu P. (1986). “The forms of capital,” in *Handbook of Theory and Research for The Sociology of Education*, ed. Richardson J. G. (Westport: Greenwood Press), 241–258.

[B5] Brown G. Michinov N. (2019). Measuring latent ties on Facebook: a novel approach to studying their prevalence and relationship with bridging social capital. *Technol. Soc.* 59:101176. 10.1016/j.techsoc.2019.101176

[B6] Brown S. (1997). Metal-recognition by repeating polypeptides. *Nat. Biotechnol.* 15 269–272. 10.1038/nbt0397-269 9062928

[B7] Cacioppo J. T. Patrick W. (2008). *Loneliness: Human Nature and The Need for Social Connection.* New York: WW Norton & Company.

[B8] Chang C. C. (2020). *The 2019 Taiwan Communication Survey (Phase Two, Year Three): The Utility and Impacts of Media Use II (D00184) [data file].* Nangang: Academia Sinica. 10.6141/TW-SRDA-D00184-1

[B9] Chen M. H. (2014). *A Study on the Relationship Between Social Capital and Citizen Participation for University Students.* Ph.D. thesis. Pingtung: National Pingtung University.

[B10] Chen X. F. (2014). *Structural Equation Modeling: The Application of Mplus.* Zhonghe District: Long & Full Computer Typesetting CO., LTD.

[B11] Chen Y.-N. K. (2016). A study of Facebook users’ social capital and political participation. *Commun. Soc.* 35 141–183.

[B12] Cohen J. (2013). *Statistical Power Analysis for the Behavioral Sciences.* Cambridge: Academic press. 10.4324/9780203771587

[B13] Coleman J. S. (1988). Social capital in the creation of human capital. *Am. J. Sociol.* 94 S95–S120. 10.1086/228943

[B14] Docherty N. (2020). Facebook’s ideal user: healthy habits, social capital, and the politics of well-being online. *Soc. Media Soc.* 6:2056305120915606. 10.1177/2056305120915606

[B15] Elin L. (2013). “The radicalization of Zeke Spier: how the Internet contributes to civic engagement and new forms of social capital,” in *Cyber Activism*, eds McCarthy M. Ayers M. D. (Milton Park: Routledge), 107–124.

[B16] Ellison N. Heino R. Gibbs J. (2006). Managing impressions online: self-presentation processes in the online dating environment. *J. Comput. Mediat. Commun.* 11 415–441. 10.1111/j.1083-6101.2006.00020.x

[B17] Ellison N. B. Steinfield C. Lampe C. (2007). The benefits of Facebook “friends:”Social capital and college students? Use of online social network sites. *J. Comput. Mediat. Commun.* 12 1143–1168. 10.1111/j.1083-6101.2007.00367.x

[B18] Eveland W. P. (2004). The effect of political discussion in producing informed citizens: the roles of information, motivation, and elaboration. *Polit. Commun.* 21 177–193. 10.1080/10584600490443877

[B19] Furnham A. Christoforou I. (2007). Personality traits, emotional intelligence, and multiple happiness. *North Am. J. Psychol.* 9 439–462.

[B20] Gefen D. Rigdon E. Straub D. (2011). An update and extension to SEM guidelines for administrative and social science research. *MIS Q.* 35 1–7. 10.2307/23044042

[B21] Gibson R. K. Lusoli W. Ward S. (2005). Online participation in the UK: testing a ‘contextualised’ model of Internet effects. *Br. J. Polit. Int. Relat.* 7 561–583. 10.1111/j.1467-856x.2005.00209.x

[B22] Gil de Zúñiga H. Jung N. Valenzuela S. (2012). Social media use for news and individuals’ social capital, civic engagement and political participation. *J. Comput. Mediat. Commun.* 17 319–336. 10.1111/j.1083-6101.2012.01574.x

[B23] Goodman N. J. (2014). “Internet voting in a local election in Canada,” in *The Internet and Democracy in Global Perspective*, eds Grofman B. Trechsel A. H. Franklin M. (Cham: Springer), 7–24. 10.1007/978-3-319-04352-4_2

[B24] Granovetter M. S. (1973). The strength of weak ties. *Am. J. Sociol.* 78 1360–1380. 10.1086/225469

[B25] Howell R. D. (1996). LISREL 8 with PRELIS2 for Windows. *J. Market. Res.* 33 377–381. 10.2307/3152137

[B26] Hu L. Bentler P. M. (1999). Cutoff criteria for fit indexes in covariance structure analysis: conventional criteria versus new alternatives. *Struct. Equat. Model.* 6 1–55. 10.1080/10705519909540118

[B27] Ida R. Saud M. (2020). An empirical analysis of social media usage, political learning and participation among youth: a comparative study of Indonesia and Pakistan. *Qual. Quan.* 54 1285–1297. 10.1007/s11135-020-00985-9

[B28] Ida R. Saud M. Mashud M. I. (2020). Persistence of social media on political activism and engagement among Indonesian and Pakistani youths. *Int. J. Web Based Commun.* 16 378–395. 10.1504/IJWBC.2020.111361

[B29] Keppel G. Zedeck S. (1989). *Data Analysis for Research Designs.* New York: Macmillan.

[B30] Kim B. Hoewe J. (2020). Developing contemporary factors of political participation. *Soc. Sci. J.* 7 7–15. 10.1080/03623319.2020.1782641

[B31] Kim Y. Chen H. T. (2016). Social media and online political participation: the mediating role of exposure to cross-cutting and like-minded perspectives. *Telemat. Inform.* 33 320–330. 10.1016/j.tele.2015.08.008

[B32] Kim Y. Khang H. (2014). Revisiting civic voluntarism predictors of college students’ political participation in the context of social media. *Comput. Hum. Behav.* 36 114–121. 10.1016/j.chb.2014.03.044

[B33] Kline R. B. (2015). *Principles and Practice of Structural Equation Modeling.* New York: Guilford publications.

[B34] Kobayashi T. (2010). Bridging social capital in online communities: heterogeneity and social tolerance of online game players in Japan. *Hum. Commun. Res.* 36 546–569. 10.1111/j.1468-2958.2010.01388.x

[B35] Kraut R. Patterson M. Lundmark V. Kiesler S. Mukophadhyay T. Scherlis W. (1998). Internet paradox: a social technology that reduces social involvement and psychological well-being? *Am. Psychol.* 53 1017–1031. 10.1037/0003-066X.53.9.1017 9841579

[B36] Lin J. H. (2017). How do you connect on facebook? the associations of facebook connection strategies with perceived social support and psychological well-being. *J. Inform. Soc.* 32 113–149.

[B37] Lin N. (2002). *Social Capital: A Theory of Social Structure and Action.* Cambridge: Cambridge university press. 10.1017/CBO9780511815447

[B38] Neale L. Russell-Bennett R. (2009). What value do users derive from social networking applications? *First Monday* 14 1–11. 10.5210/fm.v14i9.2506

[B39] Nie N. H. (2001). Sociability, interpersonal relations, and the Internet: reconciling conflicting findings. *Am. Behav. Sci.* 45 420–435. 10.1177/00027640121957277

[B40] Ostic D. Qalati S. A. Barbosa B. Shah S. M. M. Galvan Vela E. Herzallah A. M. (2021). Effects of social media use on psychological well-being: a mediated model. *Front. Psychol.* 12:2381. 10.3389/fpsyg.2021.678766 34234717 PMC8255677

[B41] Parry G. Moyser G. Day N. (1992). *Political Participation and Democracy in Britain.* Cambridge: Cambridge University Press. 10.1017/CBO9780511558726

[B42] Pew Research Center (2012). *Social Media and Political Engagement.* Available Online at: https://www.pewresearch.org/internet/2012/10/19/social-media-and-political-engagement/ (accessed May 20, 2021).

[B43] Pew Research Center (2014). *Cell Phones, Social Media and Campaign 2014.* Available Online at: https://www.pewresearch.org/internet/2014/11/03/cell-phones-social-media-and-campaign-2014/ (accessed May 20, 2021).

[B44] Pooley J. A. Cohen L. Pike L. T. (2005). Can sense of community inform social capital? *Soc. Sci. J.* 42 71–79. 10.1016/j.soscij.2004.11.006

[B45] Putnam R. D. (2000). *Bowling Alone: The Collapse and Revival of American Community.* New York: Simon and schuster. 10.1145/358916.361990

[B46] Qiu H. Z. (2005). *Structural Equation Modeling.* Taiperi: Yeh Book Gallery LTD.

[B47] Ryff C. D. (1989). Happiness is everything, or is it? Exporations On the Meaning of Psychological Well-being. *J. Pers. Soc. Psychol.* 57 1069–1081. 10.1037/0022-3514.57.6.1069

[B48] Saud M. Ida R. Mashud M. I. (2020a). Democratic practices and youth in political participation: a doctoral study. *Int. J. Adolesc. Youth* 25 800–808. 10.1080/02673843.2020.1746676

[B49] Saud M. Mashud M. I. Ida R. (2020b). Usage of social media during the pandemic: seeking support and awareness about COVID-19 through social media platforms. *J. Public Affairs* 20:e2417. 10.2196/preprints.21090

[B50] Saud M. Margono H. (2021). Indonesia’s rise in digital democracy and youth’s political participation. *J. Inform. Technol. Polit.* 18 443–454. 10.1080/19331681.2021.1900019

[B51] Steinfield C. Ellison N. B. Lampe C. (2008). Social capital, self-esteem, and use of online social network sites: a longitudinal analysis. *J. Appl. Dev. Psychol.* 29 434–445. 10.1016/j.appdev.2008.07.002

[B52] Stevenson B. Wolfers J. (2008). *Economic Growth and Subjective Well-being: Reassessing the Easterlin paradox* (No. w14282). Cambridge: National Bureau of Economic Research. 10.3386/w14282

[B53] Tefertiller A. C. Maxwell L. C. Morris D. L. (2020). Social media goes to the movies: fear of missing out, social capital, and social motivations of cinema attendance. *Mass Commun. Soc*. 23 378–399. 10.1080/15205436.2019.1653468

[B54] Trepte S. Dienlin T. Reinecke L. (2015). Influence of social support received in online and offline contexts on satisfaction with social support and satisfaction with life: a longitudinal study. *Media Psychol.* 18 74–105. 10.1080/15213269.2013.838904

[B55] Valenzuela S. Park N. Kee K. F. (2009). Is there social capital in a social network site: facebook use and college students’ life satisfaction, trust, and participation. *J. Comput. Mediat. Commun.* 14 875–901. 10.1111/j.1083-6101.2009.01474.x

[B56] Van Deth J. W. (1986). A note on measuring political participation in comparative research. *Qual. Quant.* 20 261–272. 10.1007/BF00227430

[B57] Van Deth J. W. (2016). *What is Political Participation?.* Oxford: Oxford Press, 10.1093/acrefore/9780190228637.013.68

[B58] Verba S. Schlozman K. L. Brady H. E. (1995). *Voice and Equality: Civic Voluntarism in American Politics.* Cambridge: Harvard University Press. 10.2307/j.ctv1pnc1k7

[B59] Wang H. Y. (2014). Citizen participation: the dimension of the right to express well-being. *J. Chongqing Univ. Posts Telecommun.* 26 97–100.

[B60] Williams K. (2003). Has the future of marriage arrived? A contemporary examination of gender, marriage, and psychological well-being. *J. Health Soc. Behav.* 44 470–87. 10.2307/1519794 15038144 PMC4018193

[B61] Ye Z. K. (2006). *Leisure research - Leisure View and Leisure Monograph.* Schaumburg: Creative & More INC.

[B62] Yu M. N. (2006). *Latent Varibable Models : The Application of SIMPLIS.* Taipei: Taiwan Higher education press.

[B63] Zak P. J. Knack S. (2001). Trust and growth. *Econ. J.* 111 295–321. 10.1111/1468-0297.00609

[B64] Zhang L. Zuo B. (2007). Eudaimonic well-being: a review on psychological well-being. *Adv. Psychol. Sci.* 15 134–139.

